# Social Determinants of Health Influence on Trans and Gender-Diverse People: A Qualitative Photovoice Study

**DOI:** 10.3390/bs16020265

**Published:** 2026-02-11

**Authors:** Miguel García-Hernández, María Marín-Rodríguez, Ismael Jiménez-Ruiz, José Antonio Jiménez-Barbero, María Sánchez-Muñoz, María del Mar Pastor-Bravo

**Affiliations:** 1 Faculty of Nursing, University of Murcia, 30120 Murcia, Spain; miguel.garciah@um.es (M.G.-H.);; 2 Biomedical Research Institute of Murcia Pascual Parrilla–IMIB, 30120 Murcia, Spain; 3 Mental Health Centre of Cartagena, Murcia Health Service, 30201 Cartagena, Spain

**Keywords:** social determinants of health, gender identity, transgender persons, gender-nonconforming persons, photovoice, nursing, social stigma

## Abstract

Despite the introduction of inclusive and gender-affirming approaches in healthcare, transgender and non-binary people continue to show poorer physical, psychological, and social outcomes, shaped by social determinants within historically pathologizing and stigmatizing contexts. This study used qualitative participatory action research with photovoice among seven transgender individuals residing in Murcia, Spain; data were generated through semi-structured interviews and focus group dialogue, applying the SHOWED technique to the visual and discursive narratives of the participants, and analyzed with Atlas.ti v8. Educational, employment, and healthcare contexts significantly condition well-being. Well-being was determined by the circumstances and support in which gender identity is constructed, within sociocultural environments marked by gender stereotypes, exclusion from social spaces, and fears regarding the irreversibility of certain transition steps. Reported lifetime negative events, social barriers, exclusion, and persistent questioning of identity were associated with increased anxiety, depressive symptoms, and insomnia. At the same time, the relevance of resilience and support networks also emerged during the sessions. Replicating photovoice in diverse settings may help identify social and territorial inequities and inform improvements in clinical practice, healthcare education, public policies, and legislation for transgender and gender-nonconforming people.

## 1. Introduction

Adult transgender and gender-nonconforming (TGNC) people face worse healthcare outcomes than the cis population due to stigma and pathologization (Drabish & Theeke, 2022). The Region of Murcia, in Spain, has some contextual circumstances, in terms of legislation and policies, which are central to this study. TGNC individuals present worse health outcomes, both physical, mental, and social, than cisgender population. Access to gender-affirming care and employment, or the exclusion they face, are relevant barriers (Christensen et al., 2020). TGNC individuals show unemployment rates of 46.5% for binary transgender individuals and 69.2% for gender-nonbinary people, data which is multiple times higher than the Spanish unemployment rates for the general population (Spanish Ministry of Equality, 2022).

TGNC individuals face higher risks of cardiovascular, respiratory, and endocrine diseases and mental health disorders (Rich et al., 2020). According to the American Heart Association, trans men and trans women are more likely to suffer myocardial infarction than cisgender population (Streed et al., 2021) and subsequently exhibit greater multimorbidity and overall health vulnerability (Rich et al., 2020). Despite more comprehensive and inclusive care approaches focused on social determinants of health (SDOHs) aimed at creating safe, equitable environments for historically marginalized populations, healthcare for these individuals remains conditioned by a history of pathologization (Christensen et al., 2020). Even with regulatory advances, such as the removal of trans identities as mental disorders in the Diagnostic and Statistical Manual of Mental Disorders (DSM-5) and in the International Classification of Diseases (ICD-11), structural barriers to accessing care persist. These aspects include insufficient staff training in gender diversity and outdated or inadequate protocols that perpetuate stigma and symbolic violence (Kanamori & Cornelius-White, 2016; Winter et al., 2016). TGNC people tend to avoid healthcare services, experience delayed diagnosis, and experience the reproduction of institutional violence (Goulding et al., 2023). As de Blok et al. (2019) and Safer (2021) note, there is an urgent need to implement clinical follow-ups that simultaneously consider gender identity and biological sex, including screening and risk assessment for conditions such as prostate cancer in trans women or breast and uterine cancer in trans men (de Blok et al., 2019; Safer, 2021). Minority stress theory provides an explanatory framework for these phenomena, presenting that stigma, discrimination, and chronic victimization generate a sustained emotional and physiological burden that directly affects health outcomes (Larrucea-Iruretagoyena & Orue, 2021; Streed et al., 2021). Therefore, it is essential to complement the analysis of SDOHs with an intersectional perspective (Crenshaw, 1989), which reveals how different axes of inequality interact and exposes the limitations of fragmented public policies (Espelt et al., 2016; Panamerican Health Organization & World Health Organization, 2025)

Participatory action research (PAR) and techniques such as photovoice enable analysis of issues including hypervisibility, social hostility, identity recognition, and symbolic representation in public spaces. In trans and queer contexts, these methods have highlighted experiences of exclusion, as well as agency for change and resilience (Christensen et al., 2020; Holtby et al., 2015; Sanz Vega et al., 2018). Furthermore, they have documented the effects of structural violence on the health of racialized trans women, particularly regarding their mental and sexual health (Gailits et al., 2022; Ussher et al., 2022).

In the Region of Murcia, the setting of the study, the legal and policy context for LGBTIQ+ equality and non-discrimination—providing specific recognition and protections for TGNC people—was strengthened with the enactment of Law 8/2016 (Region of Murcia Government, 2016). Since 2020, Murcia has also implemented a Regional Healthcare Protocol for Trans People, which sets out actions across primary healthcare, endocrinology, and mental health services, including guidance on pre-hormone therapy assessment, surgical options, and reproductive healthcare, with emphasis on the need for comprehensive training of healthcare professionals (Álvarez-Castillo et al., 2020). More recently, the Spanish parliament approved Law 4/2023, which compiles the legal framework for effective equality, including the recognition of gender self-determination and protection against stigma and discrimination (Spanish Government, 2023).

In this context, healthcare professionals—and particularly nurses—play a fundamental role in addressing the specific needs of trans people, not only in clinical care, but also in challenging stigma, promoting self-care, and transforming institutional practices towards more inclusive models (García-Acosta et al., 2024; Kanamori & Cornelius-White, 2016). Nurses are central to creating inclusive healthcare environments where trans individuals are heard and receive holistic, individualized care (García-Acosta et al., 2024). However, the existence of structural and symbolic barriers underscores the need for methodologies that incorporate identity diversity as an analytical axis and promote genuine participation (Safer, 2021).

### 1.1. Theoretical Frameworks

SDOHs constituted the theoretical framework for this study, with the aim focused on structural determinants (political context and socioeconomic position) and intermediary determinants (living and working conditions and psychosocial factors) of health (Gailits et al., 2022; Panamerican Health Organization & World Health Organization, 2025). An intersectional approach was applied in the data analysis because its connection to public policies enables the examination of simultaneous impacts and resistance to structures and systems of oppression and domination (Frías, 2022).

### 1.2. Current Study

To address this lack of evidence, we pose the following research question: How can photovoice help us understand the influence of SDOHs on the health outcomes of the TGNC adult population in the Region of Murcia, Spain? The specific objectives are: (1) to examine health perceptions and needs in relation to their social and healthcare environment; (2) to analyze the expressive and critical potential of photovoice as a tool for individual and collective empowerment; and (3) to foster participatory dialogue that highlights inequalities, generates critical awareness, and guides more inclusive public policies —led or supported by nurses.

## 2. Materials and Methods

This study was exploratory in nature, using a qualitative PAR design that combined semi-structured interviews in a focus group and the photovoice method to explore how SDOHs affect TGNC people, through their own visual and discursive narratives. This study was conducted within the TranSalud project: participatory action research for the promotion of care for transgender people in the Region of Murcia. Qualitative PAR methodologies are well suited to generating empowerment and visibility for the transgender community in healthcare settings since they enable subjective and collective expression (Christensen et al., 2020). The methodology was developed in accordance with the COREQ guidelines (Tong et al., 2007), ensuring rigor in the study design, data collection, and analysis.

### 2.1. Participants

In total, 18 people were recruited for the study, of whom 7 eventually participated (Table 1). The average age was 29.7 years; most (*n* = 5) were aged 18–25. Four identified as male, one as female, and two as gender non-binary (one identified as gender fluid). Most lived with a partner or close relatives. Five were unemployed, and six reported annual income ≤€12,000. Five of seven had secondary education, and the remainder had basic education. Although all valued support for the trans community, only three participated in LGBTQ+ associations (Gylda, Cats and Colectivo-No-Te-Prives). Even though not every participant had started medical transition, everyone had performed social transition: three of seven had not started hormonal transition—one of whom did not initiate it since they wanted to enlist in the state police—one had started it recently (less than 2 years from the project beginning), two had initiated it more than 5 years before, and one of them started medical transition more than 10 years before the study.

### 2.2. Study Setting and Recruitment

The target population for this study was TGNC individuals aged ≥18 years residing in the Region of Murcia. Sampling was intentional and non-probabilistic via social media and local LGBTQ+ associations. Once the first participants agreed to take part in the study, snowballing and referrals were employed. This strategy for recruitment was as follows: (1) simultaneous diffusion of the study and asking for participants via social media and local associations, (2) individuals who were close to the research team, (3) snowballing and participant referrals. Highly isolated or TGNC people in the early stages of their transition could not be reached through this strategy. Likewise, TGNC individuals who had no availability to attend could not participate.

### 2.3. Inclusion and Exclusion Criteria

Inclusion criteria were: (1) TGNC individual; (2) age ≥ 18 years; (3) residency in the Region of Murcia; and (4) Spanish language proficiency. Visual or cognitive disabilities were exclusion criteria. The final sample comprised seven TGNC people aged 18–55.

### 2.4. Data Collection

Data collection instruments included: a sociodemographic questionnaire with inclusive language; photovoice sessions structured according to the SHOWED method; field notes by the research team; and participant-generated photographs and texts.

The PAR comprised five flexible, dialogical cycles: C1. Identification of the internal environment (individual interviews and sociodemographic questionnaire). C2. Exploration of the external environment (individual photovoice exercise). C3. Collective understanding of the problem (presentation and group discussion of images in focus group sessions). C4. Strengthening self-care (co-creation of a meaningful learning workshop focused on care practices). C5. Participatory impact assessment (reflective analysis of the experience and identification of key elements for a replicable intervention model) (Pastor Bravo et al., 2021).

The PAR photovoice sessions in focus group meetings took place during five of the thirteen PAR project sessions (18 April, 21 April, 29 April, 6 May, and 13 May), which was the total number of PAR project sessions. M.P.-B. and M.M.-R. attended every session, adopting a facilitator role. These sessions combined individual work with collective discussion. The research team acted as facilitators, promoting horizontal relationships, a climate of trust, and shared decision-making at all stages. Group sessions were led, as stated, by M.P.-B. and M.M.-R., experienced in qualitative research and community support.

The intervention took place in settings agreed upon with participants, prioritizing safe, accessible, and comfortable spaces (eventually, all sessions took place at the University Social Centre on campus in Espinardo, Murcia, Spain, with participants’ prior agreement). To minimize transport costs or access barriers, the research team provided transport when needed.

In an initial group session, the methodology was presented, and a detailed instruction sheet (Appendix A) was provided. Each participant freely selected images and wrote a short reflective text (max. 150 words), guided by an adapted SHOWED method with the following questions: What can I observe in the photo? What is really happening? How does this relate to your life? Why does this problem exist? How does it affect my health? What do I want to communicate with this image? Based on individual, reflective work, subsequent group sessions focused on discussion and co-creation around factors conditioning their health, experiences related to access to care, and self-care, with a reflective, critical approach.

### 2.5. Data Analysis

Sociodemographic data and photovoice sessions were audio-recorded and transcribed verbatim. Photographs were catalogued for coding and linked to their corresponding explanatory SHOWED texts. Atlas.ti-V8 software was used for coding. For data analysis, the inductive-deductive procedure proposed by Fereday and Muir-Cochrane (2006) was followed.

In the first phase, each photograph was analyzed within its group context, prioritizing the narrative of the participant who authored the image. Subsequently, a cross-sectional analysis was conducted that integrated the shared meanings and the group’s emerging beliefs. SHOWED transcripts and images were read iteratively, and initial line-by-line codes were generated from the textual content, systematically contrasted with the visual elements of each photograph (visual-textual triangulation).

Coding was performed independently and in pairs by M.P.-B. (nurse, doctoral researcher) and M.G.-H. (nurse, researcher), who jointly developed the codebook. Discrepancies were discussed in biweekly consensus meetings, following a decision rule based on reflective agreement; when immediate consensus was not reached, the original data (image and text, SHOWED) was returned until a shared interpretation was achieved. A thoughtful analytical audit was performed by systematically documenting decisions and codebook reviews.

The initial codes were progressively grouped into categories and themes, with a record of the analytical process maintained. Verbatim transcriptions were returned to the participants to confirm the accuracy of the content. M.G.-H. translated the selected final fragments into English, prioritizing semantic and cultural equivalence; participants did not validate the translations due to limited English proficiency. A detailed example of the analytical process from raw data to theme construction is presented in Table 2 to illustrate the traceability between data, codes, and results.

### 2.6. Ethical Considerations

The study complied with the principles of the Declaration of Helsinki and was approved by the Ethics Committee of the University of Murcia (code 4848/2024). Participants provided written informed consent after receiving clear information about objectives, implications, and data use. Confidentiality was ensured by assigning participant codes, and audio recordings were deleted after transcription and analysis. The pronouns chosen by each participant were used consistently, in line with a respectful and gender-affirming approach. The research team informed participants that photographs would be used in an exposition after the study to raise public awareness of trans realities; participants decided whether to appear and what contextual elements to depict.

### 2.7. Rigor and Reflexivity

Methodological rigor was ensured by adhering to the criteria of credibility, transferability, confirmability, and consistency. Credibility was reinforced through the verbatim transcription of the recordings and the fidelity between the dialogues, the images produced, and the explanatory texts prepared by the participants. Portability was enhanced through explicit description of the study context and the application of clear inclusion and exclusion criteria. Confirmability was addressed by limiting analytical interpretation to empirical data (transcripts and images) and by maintaining a systematic record of the analytical process. Consistency was ensured through detailed documentation of methodological decisions, theoretical framework, field notes, and phases of analysis. In terms of reflexivity, the research team continuously examined their positionality, researcher–participant relationships, and the processes developed in situ (Berenguera Ossó et al., 2014).

The team comprised a psychologist, a mental health nurse, and three nurses, all institutionally affiliated with the same university and health system, including a doctoral student and a predoctoral researcher. These career paths, close to the field of study, were recognized as potential sources of both contextual understanding and bias. To deal with inherent participatory research power dynamics, two researchers (M.P.-B., M.M.-R.) rotated the facilitation role to reduce power asymmetries and limit facilitator dominance. Power was further addressed by positioning participants as co-researchers: they selected and discussed their own photographs during photovoice sessions, collectively set discussion priorities, and influenced analytic directions across all PAR phases (Pastor Bravo et al., 2021).

Moreover, specific strategies to mitigate bias were implemented, including independent pair coding and reflexive discussion when analytical discrepancies arose. In the case of the participant who had a prior personal relationship with a member of the research team, that member refrained from participating in the coding of the participant’s data. This circumstance was explicitly addressed in the team’s reflexivity processes to minimize potential influences arising from prior relationships.

## 3. Results

The analysis revealed six major themes describing experiences of barriers with socialization, gender identity, social stigma, and care among TGNC participants. Anonymization was a priority in presenting the results, eliminating every mention that could identify participants. Images were provided by participants themselves, obtaining their consent for the generation and reproduction. An overview of these themes is presented in Table 3; a detailed description of themes and categories is provided in Appendix B. Each theme is described in the following sections.

### 3.1. Theme 1: Discourse and Society

Participants criticized the stereotypical representation of LGBTQ+ people in literature, viewing them as distant from their own realities: “Sometimes books that deal with LGBT issues are stereotypical. They repeat the stereotype of the binary system as well as stereotypes that have been generated within the LGBT community” (P5). These perceptions were mentioned by five of seven participants, primarily referring to the need for realistic LGBTQ+ role models: “I know of very few films about trans men. It’s like super dodgy, without, of course, ending in suicide or us cutting our veins …” (P5). “If there is a transsexual woman, she is always the prostitute in the film” (P1).

Participants emphasized the importance of producing their own narratives, especially amid the rise of anti-trans speech, and advocated for diversity education from early ages:

*“When I give workshops, I don’t want to change the kids’ minds, but I just tell them one thing: I need you to respect me. I mean, (…) don’t mess with your classmates who are here in the movement or with me. Don’t do it, because there was a kid who raised his hand and said, “I’ve been insulted in this class, a gay boy,” and do you know what the others did? They laughed (…). I’m telling you about this experience because I must tell you about it; it’s been like an anti-LGBT tsunami in the classrooms”.*

(P5)

Participants called for social awareness grounded in tolerance, respect, and understanding towards LGBTQ+ people, particularly the trans community. They described social context as a constant source of pressure, judgment, and demand for external validation, with direct impacts on emotional well-being and identity. At the same time, they referred to legislative advances amid uncertainty surrounding the passage of the trans law in Spain.

*“Look, I’m an adult (…). Older people have already lived their lives; it’s about raising awareness among young people because, as they become more aware, better times will come. But if they follow in their parents’ footsteps … I’ll tell you, in my job I’ve been yelled at by a man walking with his son … “Faggot!” What is that child going to learn? Raising awareness among children, raising awareness in schools, where young minds are formed. But society doesn’t change!”.*

(P5)

### 3.2. Theme 2: Social Context

Social context and socialization experiences strongly influenced identity construction, health, and well-being. School years were decisive moments: three of seven reported negative experiences and bullying, and two of seven regretted dropping out of school.

*“In my second year of high school, I had a very hard time with bullying …, going to class every day, having to see people who didn’t … and I had a very hard time with that, and I also have high intellectual abilities … So, I also had a lot of problems with teachers because of that, because I was very distracted in class. I would fall asleep, start drawing, or do something like that. I had problems with the teachers, and I had problems with the students. That led me to feel very bad about myself, to enter a bubble, a bubble of darkness and ugly things that led me to stop studying, to literally leave my exams blank and to fail everything”.*

(P1)

Similarly, participants noted misinformation about trans identities in childhood, the absence of compulsory diversity education at school, and limited teacher awareness:

*“It’s true that since I was born, I’ve always … The thing is, when we were little, at least in my environment, I didn’t know that trans boys existed. But if there had been the same information that there is now, I would have told my parents a lot about why I had to be born a woman, that I wanted to be a man … And of course, if I made these comments now, there would be enough information to take the step from a very young age. But since that wasn’t the case, as I grew up, I assumed that it was wrong. The information society and school gave me, and then … life events, led me to assume that what I did, how I dressed, and how I acted were wrong. So, I completely conformed, I tried everything, to what was socially imposed on a woman”.*

(P8)

P5 often expressed that reactions of adolescents and the youth included questioning, limited understanding of queer realities, and a perceived rise in LGBTQ+phobic behavior.

*“They don’t see the difficulties faced by people in the LGBT movement precisely because they are outside the norm of the binary system, the heterosexual system, the traditional gender system. No, they sometimes find it very difficult to see respect; it’s just about respect, and saying, ‘I don’t care who you sleep with’. I don’t care how you feel. (…) Are you capable of respecting me just for that? (…) You can’t imagine, they get as red as your red shoes, and some of them leave the class because they can’t stand the argument. They have so much hatred … I don’t know where it comes from. At fourteen, how can you have so much hatred?”.*

(P5)

**Figure 1:**
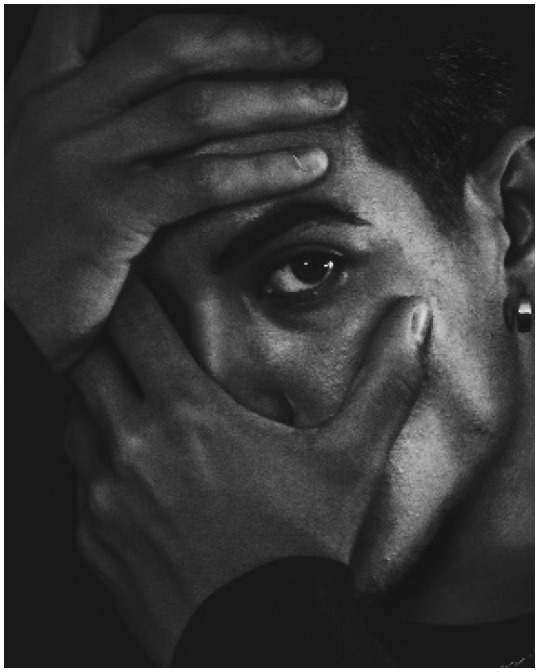
“A feeling of suffocation when I look into the eyes of those who feel calm while I try to organize my thoughts, which forces me to look away so I can breathe” (P6)—participant-generated and consent obtained.

Gender identity shaped social relationships, generating insecurities, fear of eye contact, and concerns about being too reserved—or too outgoing (Figure 1, P6).

Family relationships and acceptance during transition were particularly relevant, fluctuating from empathy and support to pressure and adverse effects on mental health:

*“I was the one who started causing them (my family) problems because I was different, and I am the one who turned out to be trans, who had problems socializing, who had a lot of issues … saying I don’t agree with …, I’m sorry, but I don’t follow your beliefs when my sister also followed exactly the same as them”.*

(P2)

*“It depends on the person (their beliefs), people, actually, and obviously, some religious people understand, and others don’t. My mother is a Christian and accepts me and, in fact, she was working in an organization to support LGBT people”.*

(P1)

Five of seven participants reported episodes of violence and discrimination, including verbal abuse, intimidation, sexism, transphobia, a perceived decline in social awareness, invisibility of asexuality, and a fear of public self-expression:

*“Personally, I am afraid, and if I don’t show myself as trans, leaving my chest exposed and letting myself be, it is because I am afraid that I might be attacked. For me right now, it’s a matter of integrity and mental health, it’s not walking down the street with that fear that because I have four piercings here and my goatee, they might attack me because I have breasts and it’s clear that my biological sex is female”.*

(P5)

Participants also expressed the importance of healthy emotional relationships and concern about sexist behavior in adolescence and youth.

### 3.3. Theme 3: Education and Employment Situation

**Figure 2:**
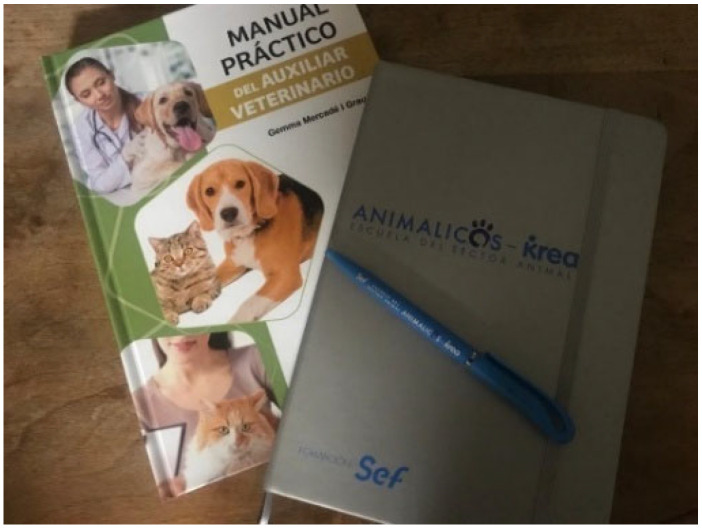
Benefits of online teaching (P2)—participant-generated and consent obtained.

Two participants expressed strong motivation to complete their training and pursue desired careers. Online teaching was highly valued for its safety, especially for those seeking to avoid negative school experiences. P2 highlighted the advantages of this method after finding a career with which he was enthusiastic about and could project into the future (Figure 2).

Moreover, they emphasized the importance of clear professional goals. Three of seven reported that they chose jobs unrelated to their vocational interests for economic stability, while pursuing their true vocation, which was linked to art, design, or artistic creation. The search for satisfying, socially impactful professions sometimes led to family conflicts when choices did not align with traditional prestige models:

*“I’m the only one who doesn’t want to study for a degree, who wants to devote myself to something other than something important, so to speak. I want to be a tattoo artist … ‘Not important’, like it’s not a degree like law or medicine … I can even have more impact than a doctor simply by tattooing three numbers on someone’s arm and them telling me that those numbers are the time their grandmother died, and that they want to carry her with them forever. So, for me, it’s not about changing someone’s life, but doing something nice for someone and being able to make a living from it”.*

(P1)

Employment situations varied among participants: P7 was unemployed due to difficulties managing diabetes at work, and most were in training (4 of 7); P3’s case was notable for participation in a training-plus-paid employment project that provided income.

### 3.4. Theme 4: Gender Identity

The narratives surrounding gender identity underscore the influence of gender stereotypes on identity construction and the division of social spaces, both in literature/film and within the LGBTQ+ community:

*“From a very young age, I told my mother that I felt like a boy, I wanted to have been born a boy, but because there were some very harsh stereotypes in my family. And at first, I associated … I was very angry about female stereotypes and associated them with being unpleasant, until finally, as I got to know women, I saw that the stereotypes were nonsense. That there were all kinds of women. And it completely changed my concept because they made me feel that … I didn’t understand some of them”.*

(P2)

The transition process raised about irreversibility, particularly among those who had not started hormonal treatments or were in the decision-making process (reported by two of seven).

*“I’m afraid, for example, even though I can’t take hormones because I want to take the police exam … Even if I could, I’m terrified by the fact that … When I had an appointment with the endocrinologist, I felt terrible for a whole week because of it, because I said something my parents had told me a lot: ‘What if I don’t want to (be a man) when I’m thirty or forty, you know?’ (what if) I want my appearance to be more feminine or I don’t know … It’s true that I have little to no femininity, but what do I know?”.*

(P8)

Using binary toilets generated divergent views: insecurity when using versus interchangeable or unisex toilet use were two points of view in that session. Concerns also extended to future parenthood during/after transition:

*“I have friends who haven’t had children and have had surgery, and then ‘oh, how I would have loved to have a child.’ But you must have that in your head: be clear about what you want to be, and how far you want to go. Because the range is so wide that there is everything”.*

(P7)

**Figure 3:**
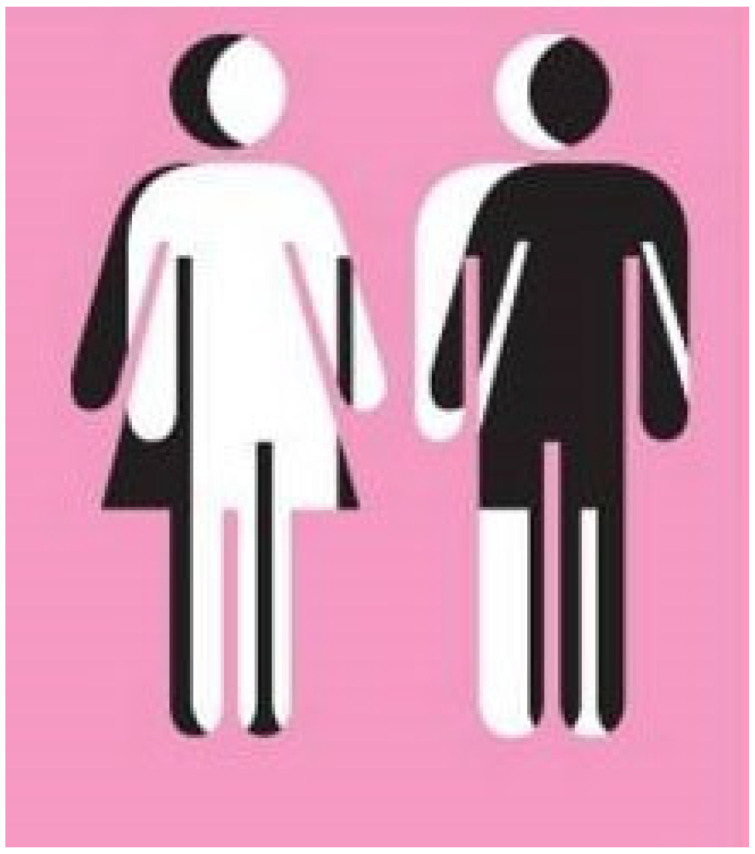
Gender questioning and the construction of one’s own identity (P8)—participant-generated and consent obtained.

Participants linked identity construction to individual experiences, conceiving gender as a gradient construct that can transcend rigid male–female categories. P8 described how questioning gender influenced identity and evoked fear of public exposure (Figure 3).

The use of affirming pronouns, deepening self-awareness, and the freedom to transition without surgery were valued by three of seven participants. Some expressed body pride without modification, while others were satisfied after surgery (e.g., P7, who underwent vaginoplasty).

*“I don’t know if you know him from TikTok, but he posts a lot about his chest. He has been on hormones for quite some time and doesn’t want to have surgery. He’s also super proud of it, and he goes to the beach and shows it off naturally. He’s a guy with boobs, and he’s so proud of it. Like the vast majority, almost all trans guys are proud of their pussy, that’s what they say. They say, ‘I’m a guy with a pussy!’ and they’re so happy”.*

(P8)

### 3.5. Theme 5: Health and Healthcare

**Figure 4:**
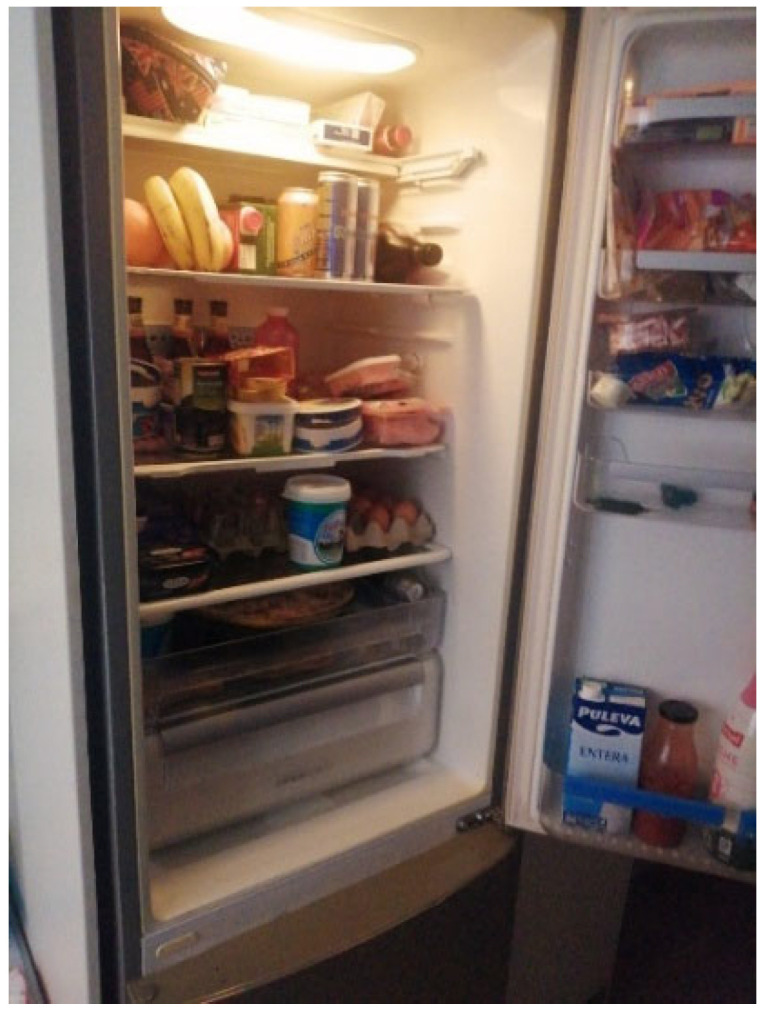
Food enjoyment and the difficulties with self-control (P7)—participant-generated and consent obtained.

Food was frequently mentioned, with a duality between enjoyment (reported by two of seven) (Figure 4) and difficulty maintaining a varied, balanced diet, given the prevalence of eating disorders (EDs) (reported by three of seven):

*“For me, it’s the opposite when it comes to weight, because … well, the issue of eating disorders and so on … months ago I wasn’t eating anything and now I weigh between 46–47 kilos, which is already low. A few days ago, I weighed myself, and I was 42. I stopped eating because I didn’t like my body, so … it’s complicated”.*

(P1)

**Figure 5:**
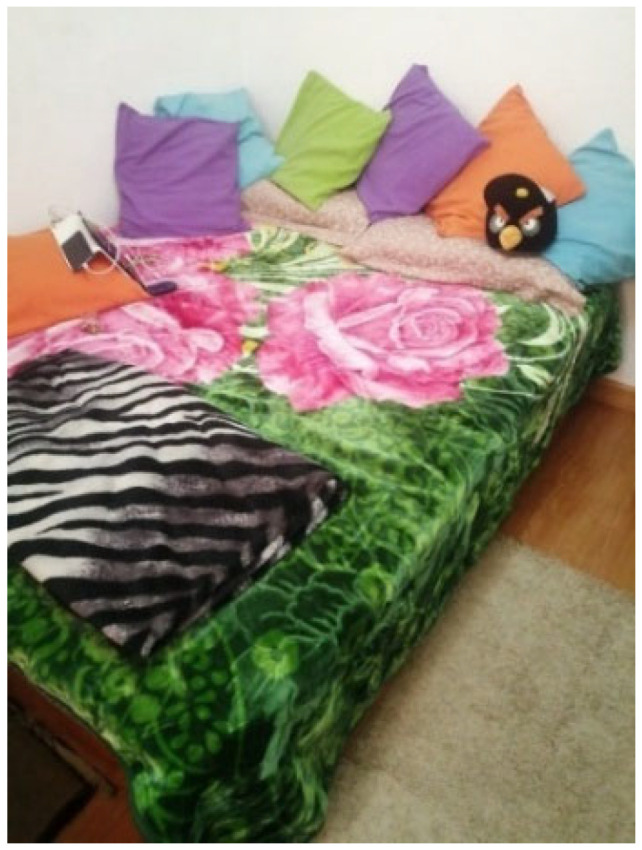
“The bed seduces me, it traps me” (P7)—participant-generated and consent obtained.

Participants discussed unhealthy habits such as weed consumption, problematic video game use, technology dependence, smoking, caffeine, sedentary lifestyles, and sleep problems as barriers to self-care (Figure 5).

**Figure 6:**
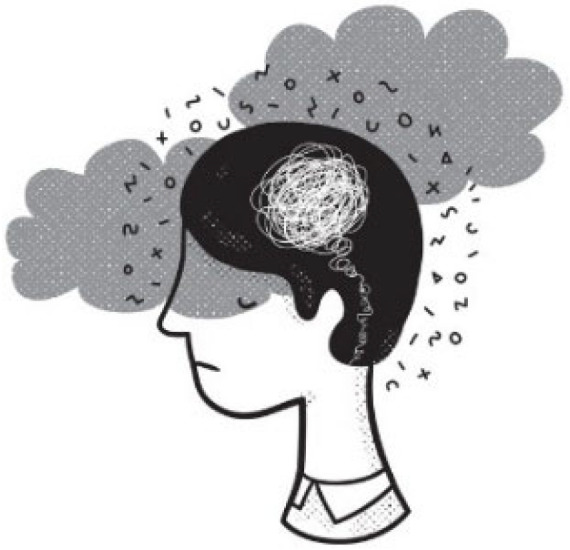
Mental health and intrusive thoughts (P8)—participant-generated and consent obtained.

Mental health was central to participants’ dialogues: anxiety, self-harm, suicide attempts, borderline personality disorder (BPD), eating disorders, and insomnia. Anxiety–insomnia links were exacerbated by nighttime electronic device use, as was noted by two of seven participants. In his photovoice, P8 expressed stress and overwhelm from intrusive thoughts and “overthinking” (Figure 6).

Regarding BPD, participants highlighted compulsive/impulsive behaviors. Experiences with EDs included school-age onset, body dysmorphia, fasting, excessive exercise, and social pressure for thinness alongside the importance of validating all body types: “(We can) raise awareness among people about how important it is not to comment on other people’s bodies and not to promote imposed standards and stereotypes” (P8).

Healthcare experiences ranged from positive private-healthcare encounters to difficulties accessing gender-affirming services and pathologizing treatment in the public system:

*“But the first thing they told me was I was disturbed, that I had an identity disorder. It’s a lottery to be able to take hormones. You had to have the piece of paper saying you were disturbed”.*

(P7)

Participants denounced the shortage of resources in public healthcare and called for more mental health professionals. They emphasized mental healthcare, asking for help, the benefits of psychological therapy, reconnecting with nature, and the role of pharmacological therapy in recovery and mental well-being. Accounts reflected positive reinforcement after overcoming difficult episodes and resilience.

*“(Working with) Emotions, right? How I feel, what I feel here now, everything I’ve been carrying in my backpack since childhood, right? It has helped me a lot to become aware of many things about myself and sometimes to know how to get out of the loops, those you get into with recurring thoughts”.*

(P5)

Support networks, peer support, and mutual advice were seen as vital: “Friendship and what that implies… Because of laughter, the ability to share emotions, it has a lot to do with mental health, and the physical well-being of a group walk” (P5).

**Figure 7:**
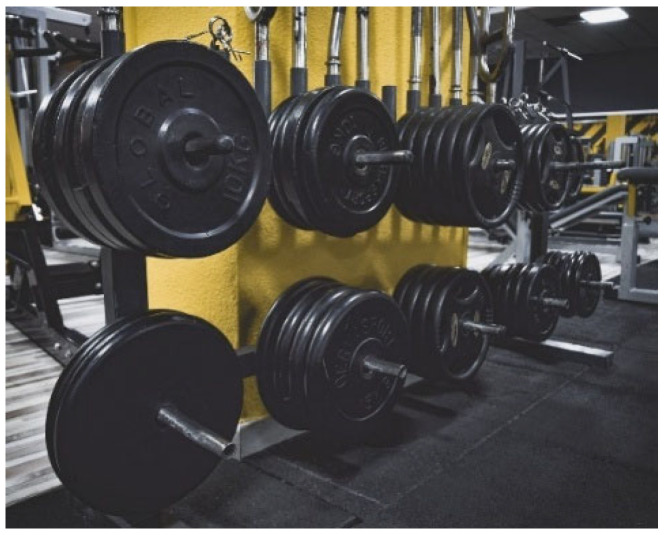
Finding what brings you peace (P6)—participant-generated and consent obtained.

Participants valued time management for self-care and meditation, and they also highlighted physical activity (e.g., hiking and exercise) to channel anxiety, escape reality, and focus on the present (Figure 7).

Participants also discussed artistic expression as motivation and as a means for awareness-raising: “Furthermore, I believe that when it comes to acting, you obviously transform yourself into a different person, and it is somewhat representative of what most of us in this group have done: transform ourselves, so to speak, into a different person” (P1).

### 3.6. Theme 6: Project Evaluation

In the final session, five of seven participants reflected positively on photovoice sessions for fostering unity and strengthening the trans community:

*“What stands out most for me is that I know there are people who listen to me, and I feel heard, I mean. And that’s very important to me because I tend to keep things to myself, ruminate a lot, and hold everything in until I explode, and so these things (the project) also help me let things flow and let go, let go a little. I feel understood and listened to, and I like that”.*

(P1)

*“I think the debate here is very enriching, and at the same time it has been a way of expressing yourself with people who … I don’t know, it’s something different. I think that as a group, being all trans, we have gone through similar things in that respect, even though in other respects we are very different, but I think we can share quite a lot”.*

(P8)

**Figure 8:**
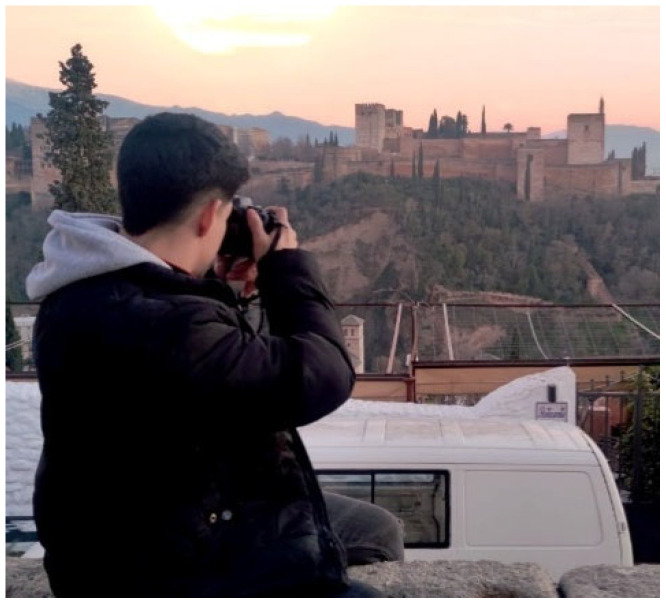
Photography helps me challenge myself (P6)—participant-generated and consent obtained.

Participants welcomed arts-based methods as channels to express experiences: “Photography helps me challenge myself to express my ideas, and although it is still a big challenge, I don’t give up” (P6, Figure 8).

Participants, at the same time, during the sessions highlighted that even though they had different experiences and backgrounds, the project had permitted them to share experiences and to realize that they had shared similar experiences:

*“So far, the session that I liked the most by far is this one (third photovoice session). But because I’ve seen it a little more familiar. I do not consider that, when the others are here, it is less familiar; I think it could be carried out well. I think it (this session and this project) is one that enriches us all, because perhaps we have exposed in some way some problem of ours, or, based on one of his, one of mine, and one of his has come out. I think that together we have been able to give ourselves a solution, not a solution, but a different perspective, or how we can help you, what we have done to solve that problem. And I think that the debate here is very enriched, and at the same time it has been a way of expressing yourself with people who … I don’t know, it’s something different. I think that the group, being all trans, we have gone through similar things in that aspect, although then in other aspects we are very different, but I think we can share a lot”.*

(P8)

## 4. Discussion

The results show that SDOHs, particularly those related to educational trajectories, employment opportunities, and healthcare access, substantially condition the physical, mental, and social well-being of TGNC people in the Region of Murcia. This is consistent with the study aims, minority stress theory, and evidence showing that stigma-related stressors operate through both psychosocial and material pathways (Larrucea-Iruretagoyena & Orue, 2021). Conceptually, our findings illustrate how structural conditions and stigma operate across life domains and how participants mobilize protective resources, including peer support and art-based expression (Holtby et al., 2015; Sanz Vega et al., 2018).

### 4.1. Mapping Empirical Themes onto Theoretical Frameworks

Participants’ critique of stereotyped LGBTQ+ representation in literature and film (e.g., trans narratives ending in suicide; trans women portrayed as sex workers) reflects distal stigma operating through cultural narratives that constrain recognition and reinforce devaluation. This aligns with minority stress accounts of how stigma atmospheres translate into chronic stress exposure (Larrucea-Iruretagoyena & Orue, 2021; Meyer, 2003; Ussher et al., 2022). At the same time, participants emphasized the perceived escalation of anti-LGBTQ+ discourse among youth, which resonates with photovoice’s empowerment and advocacy goals (Sanz Vega et al., 2018). An intersectional reading highlights that these stigma dynamics are not uniform; participants located backlash and misunderstanding particularly within classrooms and youth cultures, suggesting variation by institutional context and cohort (Bowleg, 2012; Crenshaw, 1989).

Socialization experiences strongly shaped identity construction and well-being, with school years described as decisive; bullying, misinformation, and insufficient teacher training were linked to withdrawal, lowered self-worth, and disrupted education. Such narratives map onto proximal minority stress pathways, such as expectations of rejection, hypervigilance, and concealment, and are consistent with evidence linking discrimination to adverse mental health outcomes in TGNC populations (Cooper et al., 2020; Meyer, 2003). Fear of public expression and safety concerns demonstrate how stigma climates are perceived as threats, leading to avoidance. Family relationships varied from protective (acceptance) to harmful (pressure or conflict), aligning with the literature on social support as a protective factor against minority stress (McCann et al., 2021; Scheim et al., 2024).

Education and employment narratives picture SDOH mechanisms: participants valued online learning as a “safer” environment that mitigates face-to-face discrimination; some selected jobs for economic stability while pursuing artistic vocations, and employment instability intersected with health management. These findings are consistent with Spanish evidence documenting barriers in social and labor market integration for trans people (Spanish Ministry of Equality, 2022) and broader research linking TGNC stigma to economic vulnerability and workplace discrimination (Granberg et al., 2020; Lewis et al., 2021). Recent Spanish clinical–public health discussions emphasize that implementing trans-inclusive care also depends on employment and educational environments that reduce stigma and facilitate access (Bermúdez-Pozuelo et al., 2024). From an SDOH perspective, these results reflect how structural opportunities (education and labor markets) shape material security and, consequently, health (Committee on Educating Health Professionals to Address the Social Determinants of Health et al., 2016; Marmot, 2005).

The participants described identity construction within cultural and social contexts shaped by gender stereotypes and segregated spaces, alongside fear and uncertainty about the irreversibility of transition-related decisions, consistent with prior research on transition-related stress and contextual influences (Cooper et al., 2020; Scandurra et al., 2019). Conversely, affirming pronouns and autonomy in transition processes (including freedom to transition without surgery) were associated with well-being, reflecting protective processes and resilience described in the literature (McCann et al., 2021; Scandurra et al., 2019). Divergent experiences with binary toilets further highlight how institutional infrastructures can produce stress as participants actively negotiate safety and visibility, given that experiences vary by embodiment and context (Bowleg, 2012; Crenshaw, 1989).

Participants reported ED difficulties, unhealthy habits (e.g., substance use, sleep problems, and sedentary lifestyle), and high mental health burden (anxiety, insomnia, self-harm, suicide attempts, and BPD-related behaviors). This pattern is consistent with evidence linking chronic stigma-related stress to mental health vulnerability and health-risk coping behaviors, as well as elevated multimorbidity among TGNC populations (Cooper et al., 2020; Larrucea-Iruretagoyena & Orue, 2021; Scheim et al., 2024). Healthcare experiences ranged from positive private psychology encounters to barriers in public gender-affirming care and accounts of pathologizing treatment and resource shortages, consistent with evidence that stigmatizing protocols can lead to avoidance of care (Lo & Horton, 2016; Safer, 2021; Winter et al., 2016). In line with the most updated international standards emphasizing non-pathologizing, person-centered, and accessible gender-affirming care, these accounts underscore the need for system-level implementation and healthcare professionals’ training (Coleman et al., 2022).

Participants’ positive evaluation of photovoice—feeling heard, strengthened group cohesion, and facilitated expression—supports photovoice as an empowerment-oriented approach and highlights protective social capital (peer support and mutual recognition) that can act as a protective factor for minority stress (Holtby et al., 2015; McCann et al., 2021; Sanz Vega et al., 2018). While photovoice is widely used for empowerment and advocacy, recent syntheses suggest that effects vary by outcome and context, reinforcing the importance of linking photovoice outputs to concrete implementation channels and stakeholders (Catalani & Minkler, 2010; Halvorsrud et al., 2022).

### 4.2. Mechanisms Operating in Murcia

Our data were collected between April and May 2022, in a regional context shaped by Murcia’s specific legal and clinical frameworks: Regional Law 8/2016 establishes rights to equality and protection from discrimination based on sexual orientation and gender identity, including explicit recognition for trans people; and the Region’s Protocol for Trans Population Access to Healthcare was issued in 2020. As local healthcare protocol for transgender healthcare was approved in December 2020, limited implementation was undertaken while the empirical work was performed, and few implications for TGNC participants were noted since their medical transition took place, in most cases, prior to its approval (Álvarez-Castillo et al., 2020; Region of Murcia Government, 2016).

Several mechanisms may help explain the observed patterns locally. First, participants’ accounts of inconsistent access and pathologizing interactions may reflect implementation gaps during the transitional period—protocol presence does not automatically translate into practice change without adequate training, resources, and accountability (Álvarez-Castillo et al., 2020; Coleman et al., 2022; Safer, 2021). Secondly, despite legal protections, participants described persistent stigma and perceived backlash in everyday settings (especially schools), suggesting a lag between legal norms and social norms (Carlstrom et al., 2021; Region of Murcia Government, 2016). Third, education-to-employment trajectories described by participants—economic compromise, avoidance of hostile environments, and reliance on “safer” modalities like online training—may reflect local opportunity structures that intensify vulnerability when combined with minority stress and mental health burden (Lewis et al., 2021; Panamerican Health Organization & World Health Organization, 2025; Spanish Ministry of Equality, 2022).

### 4.3. Implications for Practice and Policy

Building evidence on the impact of SDOHs on TGNC health and well-being enables healthcare professionals—especially nurses—to prioritize outcomes and generate knowledge to inform protocols and clinical practice guidelines. Nurses play a crucial role in caring for TGNC individuals, as they can lead interventions across different settings (hospitals, primary healthcare, and schools) (García-Acosta et al., 2024). From empirically identified findings (especially fears of revealing their identity, pathologizing experiences in treatment, and avoidance of public spaces), the following prioritized interventions are proposed.

#### 4.3.1. Short-Term Actions (0–6 Months)

Firstly, since participants described fears about revealing their identity and avoidance of healthcare systems because of stigma, confidential, disclosure-safe entry points in primary care and mental health should be implemented through initial anonymous screening/triage, as should confidential liaison to avoid exposure and stigma; this intervention might preferably be nurse-led to support nurse–patient relationships and create safe spaces. Moreover, since narratives evidence pathologizing interactions and lack of knowledge about updated protocols, we propose the implementation of rapid training micro-modules for key staff (primary care, mental health, and endocrinology) in the form of short mandatory training on pronouns, non-pathologizing language, trauma-informed care, and referral intervention aligned with current standards. Finally, using participant-generated (and consented-to) photovoice outputs for targeted training and stigma reduction for both healthcare workers and society at large, involving community associations and municipalities.

#### 4.3.2. Medium-Term Actions (6–18 Months)

Considering bullying experiences, misinformation, and the lack of teacher support as decisive factors in discomfort and school dropouts, we recommend that regional education authorities, heads of schools, nurses, and educational counselors improve school-based diversity education programs and strengthen anti-bullying responses through TGNC-inclusive curricula and teacher training. Moreover, regional mental health services, primary care, and nursing care supervisors could promote access to affirming psychological care and ensure that healthcare workers are adequately informed about local protocols for trans healthcare so that TGNC have access to affirming mental health support and coping strategies. With respect to employment, Spanish Act 1026/2024 of October 8th obligates employers with more than 50 workers to have a work plan to support equality and anti-discrimination against LGBTQ+ populations; in this context, regional employment services should ensure that these measures are implemented and barriers to TGNC employability are addressed (Spanish Government, 2024).

#### 4.3.3. Long-Term Actions (+18 Months)

Given that participants noted delays, discontinuities, and inequalities in specialized care, it is recommended that regional health authorities, hospital supervisors, and community representatives establish routine audits (wait times, referral completion, and patient experience) and quality indicators for endocrinology, plastic surgery, and primary care services. Finally, regional and state policymakers, together with cultural institutions, schools, and community associations, should make long-term investments in public campaigns and community-led cultural production to make TGNC lives visible and to move beyond stereotypes.

### 4.4. Limitations of the Study and Areas for Future Research

As this was a qualitative study, the results are not generalizable beyond the participants’ contexts. PAR requires substantial participant commitment, resulting in limited participation (one focus group; seven participants; attendance fluctuated across sessions, with a minimum of four due to COVID-19-related health problems), which stands out as the main limitation of the study. As recruitment was made through social media, associations, and snowballing from participants, selection bias may be present in terms of isolated TGNC people or individuals in the first steps of their transitions, who are not involved in associations nor have publicly identified themselves as trans. Translation risks (nuance) are also present, even though verbatim transcription was performed, and final reports were translated. Potential information bias may stem from a participant being related to a research team member, as well as from facilitation effects arising from team positionality. Similarly, unidentified confounding variables (e.g., pre-existing relationships and shared educational backgrounds) may have influenced discourse during the study. These limitations have been addressed through data triangulation across instruments, ensuring the faithful representation of participants’ accounts, and through reflexivity.

The empirical work results and analysis have underscored the need to implement future work in the form of multi-site comparative PAR to address contextual differences, the inclusion of less-connected TGNC people and the TGNC elderly, and participatory co-analysis with community researchers, involving associations and experts who accompany TGNC individuals.

## 5. Conclusions

The data results showed that SDOHs affect the physical, mental, and social health of transgender and non-binary people, who experience discrimination that intensifies barriers to employment, training, and healthcare. Although regulations and protocols exist and professionals show improvements in their treatment, stigmatizing practices persist, highlighting that the mere existence of regulations does not guarantee health equity unless they are accompanied by effective, context-sensitive, and community-based implementation. Photovoice proved effective in making experiences visible, fostering critical thinking, and promoting “silent empowerment” through participatory dialogue.

The study underscores the usefulness of participatory nursing methodologies in historically silenced communities. It suggests replication across regions to identify territorial inequalities and improve clinical practice, public policies, and current legislation. Advancing health equity requires adapting services to the real needs of the trans community.

## Figures and Tables

**Table 1:** Description of the study participants.

Variable	Category	*n*
Age (years)	18–26	5
	46	1
	55	1
Gender	Female	1
	Male	4
	Nonbinary	1
	Nonbinary, gender fluid	1
Profession/Occupation	Student	4
	Equality promoter; executive	1
	Delivery man; photographer	1
	Retired	1
Employment status	Unemployed	5
	Eventual contract	1
	Undefined contract	1
Average annual income	0–12,000€	6
	12,000€–20,000€	1
The importance of having a strong trans community	A lot	6
	Quite	1
LGBTQ+ association membership	Cats	1
	No-Te-Prives	1
	Gylda	1
	No	4
Years since medical transition	No transition started	3
	≤2 years	1
	>5 years	2
	>0 years	1
Level of education	Basic	1
	Medium	6
Who they usually live with	Partner	3
	Partner & child	1
	Father	1
	Mother & sister	1
	Partner, parents-in-law & brother-in-law	1

**Table 2:** Example of coding analysis.

Raw Photovoice Caption	P2-SHOWED Text	Line-by-Line Initial Codes	Grouped Codes	Theme
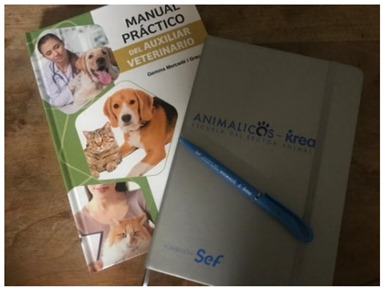	(P2): “Well, in this photo, you can see the veterinary assistant guide with the notebook and pen that they give you in the course. Because it’s a course I have been wanting to do for a long time, and it means something to me that I want to dedicate myself to. And I spent a lot of time chasing it because it’s something I really want to do. And the course is fulfilling me a lot, and I really feel that I could perfectly dedicate myself to it, even if I thought I might be a little impressed by the operating room. So far, I haven’t had any problems. And that is a very big advance for me, (…), I had not been able to (do it) because these are courses that only existed paying a lot of money until I did the first course that same year.”	Online teaching	Training education	Education and employment situation
		Online teaching: advantages—comfort		
		Motivation for practical methodologies		
		Study motivation for achieving work goals		
		Motivation for keeping their training		
		The importance of choosing jobs for their social recognition	Profession	
		Need to be productive	Mental health	Health and healthcare
		Family pressure: role of family models comparison	Social relationships	Social context
		Family pressure: finish studies		
		Feeling not enough		

**Table 3:** Themes and categories.

Theme	Category
Discourse and society	Culture and literature
	Politics and trans people
	LGBTQ+ community references
	Trans discourses
Social context	School years
	Social relationships
	Situations of violence and discrimination
Education and employment situation	Training education
	Profession
Gender identity	Gender stereotypes
	Conflicts and fears
	Identity construction
	Mental health-favoring aspects
Health and healthcare	Nutrition
	Unhealthy habits
	Mental health
	Healthcare and access
	Mental healthcare and support networks
	Self-care
Project evaluation	

## Data Availability

The original contributions presented in this study are included in the article. Further inquiries can be directed to the corresponding author.

## Abbreviations

The following abbreviations are used in this manuscript:

SDOHs	Social determinants of health
TGNC	Transgender and gender-nonconforming
PAR	Participatory action research
DSM-5	Diagnostic and Statistical Manual of Mental Disorders, Fifth Version
ICD-11	International Classification of Diseases, Eleventh Version
EDs	Eating disorders
BPD	Borderline personality disorder

## Appendix A. Photovoice Instructions Sheet

Step 1: Collecting photographs

To conduct this dynamic, we have to collect images or photographs that give us information about our perception of our own health, self-care, and barriers or factors that intervene in our health.

For the selection of these images, it is important that we ask ourselves the following questions before choosing them:

- What can I see in the photo?
- What is really happening in the photo?
- What does what I observed mean in my life?
- Why does this problem exist? How does it affect my health (physical, mental, emotional/social)?
- What do I want to communicate through this photograph?

This last question will help us to conduct the second step. Once these questions have been asked, each participant will evaluate the selected image, and if they finally choose it, they will move on to step 2.

Step 2: Adding Self-Explanatory Text

After selecting the images, each author must accompany each photograph with a short text (no more than 150 words) that is able to represent and explain the chosen theme and what each author wants to convey with the photograph. These texts allow us to understand the meaning each image holds for each author and to relate it to the central aspect: health and self-care.

Aspects which need to be considered:

Photographs will be compiled after the Hand Mapping sessions and prior to the second group session (8 April 2022).

They can be photographs or images that you previously had or new ones.

Images can be sent by email to the following address: or to the researchers via WhatsApp V2.22.8 until 7 April 2022, 3 p.m.

Some rules:

Since the photographs may be displayed on the website and in the exhibition, nude photographs, photographs documenting illegal activity, and photographs that endanger the safety of the person taking the photograph will not be taken. If the faces of third parties are visible, the recognizable individuals in the photograph must have signed a consent form for the photo to be published on the website or printed for exhibition.

## Appendix B. Codebook

**Table A1:** Codebook.

Theme	Category	Subcategory
Discourse and society: Perception of the influence of social beliefs and representation in the lives of trans people and their own representation.	Culture and literature: The influence of culture, literature, and art on the social context of trans people and their representation within them.	-
	Politics and trans people: political awareness of TGNC people.	
	LGBTQ+ community references: The importance of associations and having community leaders in the social sphere.	
	Trans discourses: Beliefs and social perceptions about TGNC people.	
Social context: Experiences and experiences derived from the socialization process.	School years: Experiences during school that influenced their identity construction.	High school experiences: A set of experiences and influences on childhood and adolescence in the school environment.
		Current reactions in the youth: Adolescent and youth perceptions of TGNC people.
	Social relations: Influence and experiences of interpersonal relationships.	Affective relationships: Experiences in romantic or romantic relationships due to their gender identity.
		Interpersonal relationships and insecurities: emotions and concerns in contact and dealing with other people.
		Relationship with family: Family experiences throughout life and on gender identity in terms of support, rejection, or felt pressure.
	Situations of violence and discrimination: Experiences of violence, discrimination, and transphobia in different spheres of life.	-
Education and employment situation: Perception of expectations and work situations, training, and economic situation.	Training education: perception of received education in terms of inclusion/exclusion.	-
	Profession: Experiences related to the current work environment and future job expectations.	
Gender identity: Influence of gender identity on the lives of participants.	Gender stereotypes: Influence of social mandates, roles, and gender expression in society, in general, and in the LGBTQ+ community, in particular.	-
	Conflicts and fears: Difficulties and dilemmas that arise in relation to gender identity.	
	Identity construction: Experience of the process of affirming gender identity.	
	Mental-health-favoring aspects: Circumstances that promote or demonstrate mental well-being with gender identity.	
Health and healthcare: Management strategies in healthcare from a biopsychosocial approach.	Nutrition: Dietary habits, as well as management of pathologies that determine these habits.	
	Unhealthy habits: Unhealthy actions taken by participants, such as a sedentary lifestyle, toxic habits, etc.	
	Mental health: Mental healthcare, ranging from mental health disorders to positive aspects and advice.	Mental health disorders: The implications of mental health issues or disorders for healthcare and their own life, such as anxiety, self-harm and suicide attempts, BPD, eating disorders, and insomnia.
	Healthcare and access: Perceptions of the healthcare system, perceived experiences of professionals, and vision of the role of healthcare professionals.	
	Mental healthcare and support networks: Developing skills and strategies for mental healthcare and the influence of having a supportive social environment.	
	Self-care: Strategies for taking care of one’s own health, as well as obstacles to implementing them.	Meditation and relaxation: Assessment of meditation and relaxation as self-care strategies.
		Physical activity: Physical exercise and related healthy habits.
		Art: Aspects of art and artistic expression that influence trans people.
Project evaluation: Participants’ perceptions of the project’s and photovoice’s influence on their lives.	-	-
